# Surgical Management of Pediatric Acetabular Fractures: A Retrospective Study of 14 Rare Cases

**DOI:** 10.1111/os.70244

**Published:** 2026-01-19

**Authors:** Guy Romeo Kenmegne, Ziming Zhang, Rui Zeng, Gang Ma, Sheqiang Chen, Qiyan Zhou, Kai Zeng, Yuqing Wang, Wentong Zhao, Jiafu Miu, Yilan Liao, Shicai Fan

**Affiliations:** ^1^ Department of Traumatology, Orthopaedic Surgery Center The Third Affiliated Hospital of Southern Medical University Guangzhou China

**Keywords:** growth plates, pediatric acetabular fractures, premature physeal fusion, triradiate cartilage

## Abstract

**Background:**

Acetabular fractures in children are extremely rare, accounting for approximately 1%–4.6% of all pediatric fractures. Due to their rarity, literature on these injuries is limited, with only a few reported cases. The primary objective of this study was to present a series of uncommon pediatric injuries, outline our management approach, and demonstrate that even patients undergoing delayed surgical intervention can achieve favorable clinical outcomes.

**Materials and Methods:**

This retrospective study reviewed records of skeletally immature patients with traumatic acetabular fractures treated at our institution. Patients were surgically treated with open reduction and internal fixation through lateral rectus abdominis approach; follow‐ups included radiological assessment of bone union and internal fixation integrity. Postoperative reduction was evaluated using Matta's criteria, while functional outcomes were measured via the Modified Merle d'Aubigné and Postel Method (pain, gait, mobility) and the Harris Hip Score (HHS). Complications were documented throughout follow‐up.

**Results:**

Between January 2019 and January 2025, 14 pediatric patients with acetabular fractures (five males, nine females; mean age 11.42 ± 2.24 years) were treated and followed for an average of 33.71 ± 14.41 months. Injuries resulted from falls (57.14%), car accidents (28.57%), and motorcycle/bicycle accidents (7.14% each). According to Judet and Letournel classification, fractures included double‐column (57.14%), transverse (35.72%), and anterior with posterior hemi‐transverse (7.14%). All underwent surgery, achieving bone union. The mean Harris Hip Score was 90.35 ± 5.58, with 71.42% rated excellent, 21.42% good, and 7.14% fair. The mean Merle d'Aubigné score was 17.21 ± 1.12. Mild hip pain occurred in three patients, with no other complications.

**Conclusion:**

Pediatric acetabular fractures, typically caused by high‐energy trauma, require treatment focused on optimal outcomes and anatomical reduction, even in delayed cases. This study shows that, in specialized centers, experienced surgical teams can achieve successful reduction and satisfactory results despite delayed intervention.

## Graphical abstract

This case series of 14 pediatric acetabular fractures surgically managed via open reduction and internal fixation through a lateral rectus approach demonstrates that favorable functional outcomes are achievable, even in non‐acute cases, when performed by an experienced surgical team. Follow‐up revealed significant hip function recovery.

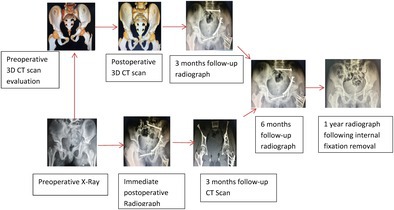

## 1 Introduction

Acetabular fractures occur when a significant force is applied between the femoral head and the acetabulum, representing approximately 0.7% of all fractures in the body [1]. Acetabular fractures are uncommon in children, making up about 1%–4.6% of all pediatric fractures [2]. It is essential to highlight that these fractures commonly result from high‐energy trauma and are frequently associated with polytrauma [3]. The acetabulum is affected in approximately 4%–20% of these patients and is often linked to pelvic ring fracture [4, 5]. If left untreated, it then heals in a deformed position, causing traumatic arthritis as a result of decreased contact area between the femoral head and acetabulum, in addition to increased local pressure and joint cartilage collapse [1].

Pelvic fractures in children differ considerably from those in adults due to variations in physiology and injury mechanisms. Due to the inherent resilience of the immature pelvis, a non‐avulsion pelvic fracture in a child should be regarded as an indicator of high‐energy trauma; as a result, the incidence of pelvic fractures in pediatric trauma patients is about half that observed in adults. This should prompt healthcare providers to consider the possibility of accompanying injuries that may pose a substantial threat to the child's survival [4]. Therefore, it is crucial for professionals in traumatology, emergency medicine, critical care, and orthopedics to understand how the patterns of acetabular fractures influence both management strategies and prognosis.

Acetabular fractures in children present unique challenges due to the complex anatomy of the pelvis. The iliac, ischial, and pubic bones are the three main centers of ossification in the pelvis through the triradiate physis, or growth plate. The growth plates of these ossification centers typically close around the ages of 16 and 18 or earlier between 12 and 14 years of age; the triradiate cartilage plays a critical role in the growth of the acetabular floor [6, 7]. Younger children, particularly those under 10 years old, who sustain injuries to the triradiate cartilage, are at a higher risk of acetabular growth disturbances. The risk of premature physeal fusion or the formation of a growth arrest bar is correlated with the pathological nature of the injury, the extent of fracture displacement, and the child's age [8].

Diagnosing acetabular fractures can be more difficult in younger children, as incomplete ossification of the periarticular structures may lead to underdiagnosis of articular surface involvement. With both the labrum and a large portion of the periarticular surface remaining unossified in younger children, plain radiographs can be misleading; on the other hand, the low incidence of hip fractures may also play an important role, leading to a lot of uncertainty in diagnosis, with severe consequences in terms of management [6, 9].

After stabilizing the patient's general condition, including hemodynamic and neurological status, the primary consideration in treatment selection is the stability of the acetabulum fracture. While stable fractures pose fewer concerns, managing unstable fractures is more challenging due to their rarity and the lack of a clear consensus on treatment approaches. It is proposed that the patient's age at the time of injury, along with the severity of the injury, is the key factor influencing the development of posttraumatic complications such as dysplasia [10]. Additionally, the timing for the operation in acetabular fracture is ideally recommended to be within 2 weeks or no more than 3 weeks in order to achieve good anatomical reduction and satisfactory clinical outcomes; however, several factors such as the polytraumatic nature of the injury and an inappropriate management plan may lead to delayed operation time. To the best of our knowledge, there is a lack of recent published consistent data on pediatric acetabular fractures, their management, a clear consensus on the operation timing and the management options. Therefore, the primary purpose of this study was to: (i) document an updated, clinically relevant case series of a rare pediatric injury; (ii) develop and evaluate a practical surgical approach tailored to the immature skeleton, balancing the goals of anatomical reduction and stability with the imperative to minimize iatrogenic growth disturbance; (iii) assess postoperative outcomes following delayed surgical management and determine whether acceptable clinical results can be achieved, thereby challenging the established principle of early intervention.

## 2 Patients and Methods

### 2.1 Study Design and Participants

The clinical data of all pediatric patients with traumatic acetabular fractures treated at our institution from January 2019 to January 2025 were retrospectively reviewed. Inclusion criteria consist of pediatric patients aged 16 or below with acetabular fracture; cases undergoing surgical treatment, and a follow‐up period of no less than 12 months. The exclusion criteria were pelvic ring fracture without acetabular involvement, patients managed conservatively, and patients who did not respond to the final assessment. Patients' demographic data, injury mechanisms, fracture type, and surgical approach were collected on an excel file from the medical record system. Acetabular fractures were divided and classified as transverse fracture, anterior with posterior hemi‐transverse, and double column fractures according to the Judet and Letournel classification [11] (Table 1). This study was approved by the Ethical Committee of our institution (date 2021‐04‐30, number 202104008), and performed in accordance with the 1964 declaration of Helsinki and its later amendments or comparable ethical standards. All patients signed written informed consent forms and agreed to publish their images for medical research.

**TABLE 1:** Overall patient information.

Parameters	Number *n* (%)
Patient number	14
Age (mean ± SD)	11.42 ± 2.24
Time from injury to surgery (days)	11.5 ± 13.36
Gender	
Male	5 (35.71)
Female	9 (64.28)
Mechanism of injury	
Car accident	4 (28.54)
Fall from height	8 (57.14)
Motorcycle accident	1 (7.14)
Bicycle crash accident	1 (7.14)
Judet Letournel classification	
Transverse fracture	5 (35.71)
Double column	8 (57.14)
Anterior column + posterior hemi‐transverse	1 (7.14)
Injury side	
Left	9 (64.28)
Right	5 (35.71)

### 2.2 Preoperative Management

A standardized preoperative protocol was implemented for all patients upon admission. This included the application of skeletal traction via the supracondylar region of the ipsilateral femur and vascular surveillance using serial bilateral lower extremity Doppler ultrasonography. Based on clinical assessment, chemical venous thromboembolism (VTE) prophylaxis, consisting of a subcutaneous dose of 0.5 mg/kg (maximum 30 mg) of low‐molecular‐weight heparin (LMWH), was initiated. This regimen is safe in pediatric patients [12] and recommended for administration within 24 h post‐injury, barring any contraindications due to high bleeding risk; additionally, sequential compression devices were recommended for all patients upon admission, unless contraindicated by concomitant lower extremity fractures or anatomical constraints preventing proper fit. Patient eligibility for surgery was categorized using the American Society of Anesthesiologists (ASA) Physical Status Classification system [13]. The surgical procedure was undertaken following confirmation of medical stability and optimal preoperative status. As part of the preoperative protocol, anticoagulant prophylaxis was discontinued 24 h prior to the scheduled procedure, and patients were placed on a strict 8‐h fast (nil per os).

### 2.3 Surgical Technique

The surgical management of pelvic ring and acetabular fractures can be achieved through either percutaneous screw fixation or open reduction and internal fixation (ORIF). The surgical option is guided by the specific fracture pattern, patient characteristics, and surgical resources. Available approaches for open surgeries include the lateral rectus abdominis, ilioinguinal and modified Stoppa approaches for anterior access, the Kocher‐Langenbeck (K‐L) or direct posterior approach (DPA) for posterior wall or column involvement, and combined anterior–posterior approaches for complex, associated fracture patterns. While our department utilizes both percutaneous and open surgical strategies, this study exclusively reports on patients managed with open reduction and internal fixation (ORIF). In all cases, the lateral rectus abdominis approach was employed, regardless of whether fixation was accomplished with plates, screws, or both.

After administration of general anesthesia, the patient was placed in a supine position on a radiolucent table. The lateral rectus abdominis (LRA) approach was the gold standard operation approach; the cutaneous landmarks for the incision are located at the outer third point of the line connecting the umbilicus and the anterior superior iliac spine (ASIS), and the inner third point of the inguinal ligament. Depending on the location, morphology, and complexity of the fracture, the incision may be extended laterally or shortened, typically ranging from 6 to 10 cm to sufficiently expose the entire hemipelvic ring.

The incision is therefore made through the skin, followed by dissection down to the deep fascia. The deep circumflex iliac artery and pubic tubercle (insertion point of the rectus abdominis) are located beneath the incision. The external oblique tendon, external oblique muscle, internal oblique muscle, and transversus abdominis muscle are then incised about 1 cm medial to the deep inguinal ring and 1 cm lateral to the pubic tubercle, moving obliquely upwards and outward. The inferior epigastric vessels and the spermatic cord are retracted cranially, while the abdominal muscles are retracted laterally and inferiorly to expose the medial window.

By retracting laterally and posteriorly, the acetabular quadrilateral area and the posterior column's medial edge can be exposed; both the middle and medial windows provide access to the medial aspect of the acetabular quadrilateral area, allowing selective exposure depending on the fracture location. The dissection continues superiorly along the acetabular anterior wall, further separating the external iliac vessels distally. From this window, dissection can be extended distally to the level of the inguinal ligament, allowing full exposure of the acetabular anterior wall and anterior inferior iliac spine. Fractures in this area can be managed relatively easily under direct vision.

**FIGURE 1:**
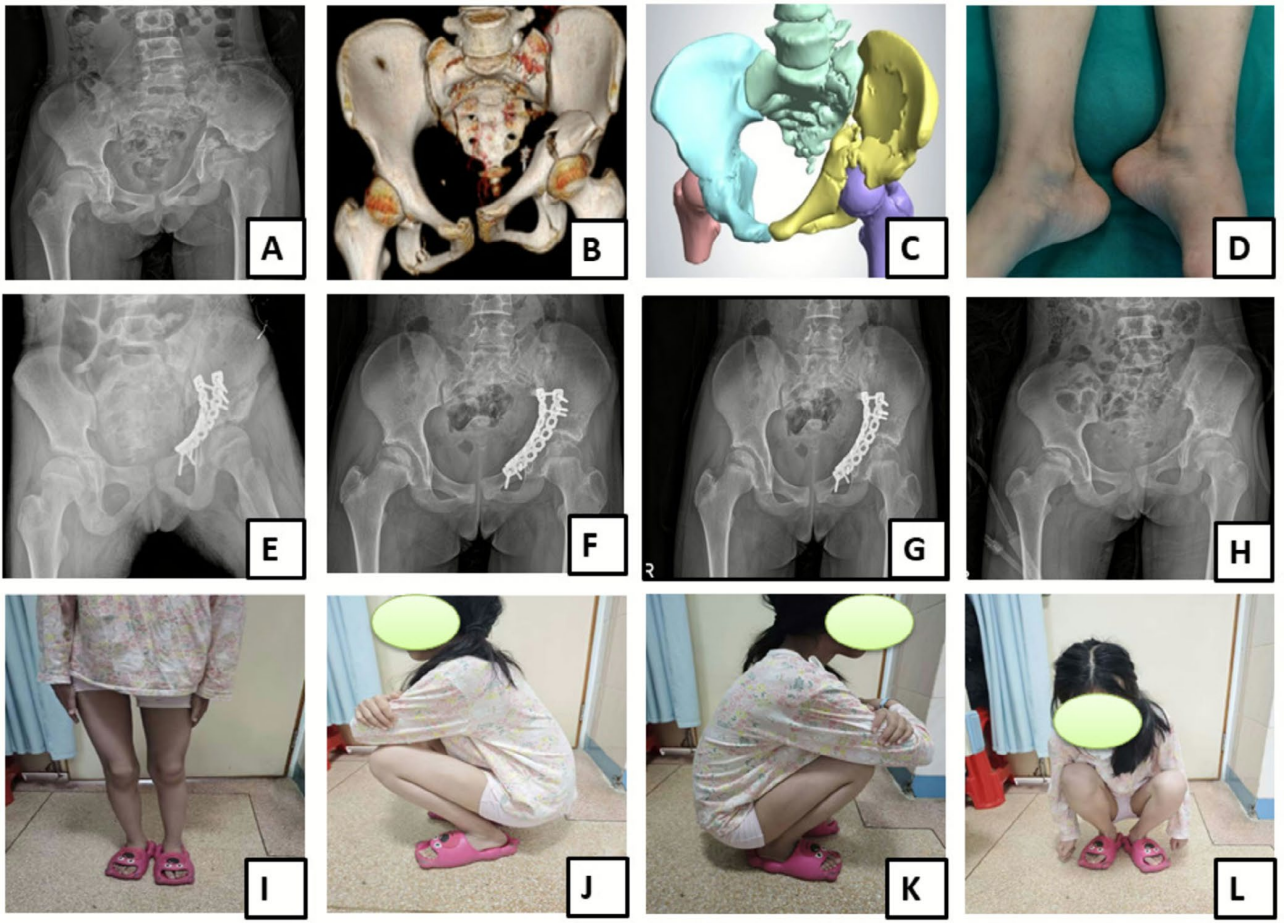
A 14‐year‐old female patient, admitted 6 weeks post‐injury of the right acetabulum following a fall from height. Post‐traumatic X‐Ray (A) and 3D CT scan (B) reconstruction image, describing the fracture lines on AP view; 3D reconstructed images (C) revealing a tilted left iliac bone with a fracture of both columns of the left acetabulum; Gross image (D) shows the discrepancy in limb length with the left leg shortened by about 3 cm; Postoperative radiographs at 1 month (E), 6 months (F), and 12 months (G) demonstrating normal fracture healing and restored joint congruence; Postoperative image (H) taken after internal fixation removal at 1 year, following complete healing; Postoperative functional outcome images (I, J, K, and L) after internal fixation removal.

As the dissection continues distally, the superior border of the obturator foramen becomes visible, thus fully exposing the quadrilateral area of the acetabulum. This area can be reduced and fixed, including the posterior column and quadrilateral area. Alternatively, a guidewire can be placed from the posterior column screw entry point, directed toward the ischial spine or lesser sciatic notch. Using this Kirshner wire as guidance, a posterior column screw can be placed under direct vision, as seen in patient number 14 (Table 2). Patients with sacroiliac joint dislocation were treated surgically with a cannulated screw (Figure 1); ideally, the robot‐assisted technique was used whenever the fracture was fixed using a cannulated screw.

**TABLE 2:** Specific patient information: demographic and clinical data.

Cases	Gender	Age	Mechanism of injury	Fracture types	Operation approach	Fixation approach	Follow up duration (months)	Matta criteria	Harris Hip Score (HHS)	Merle d’ Aubigné score
1	Female	10	Car accidents	Double column, right sacroiliac joint dislocation	LRA	Anterior column screw	51	< 1 mm	95	18
2	Male	9	Fall from height	Double column, bilateral Denis II sacral fracture	LRA	Reconstruction plate	55	8 mm	97	18
3	Male	7	Fall from height	Double column	LRA	Anterior column screw	43	< 1 mm	90	17
4	Female	13	Car accidents	Double column, unilateral sacroiliac joint diastasis, sacral fracture (Denis II), pubic symphysis injury	LRA	Reconstruction plate	39	6 mm	86	16
5	Female	10	Car accidents	Double column, unilateral sacroiliac joint dislocation, pubic symphisis diastasis.	LRA	Reconstruction plate	56	< 1 mm	92	18
6	Male	11	Fall from height	Transverse fracture, pubic symphysis diastasis	LRA	Reconstruction plate	31	< 1 mm	95	18
7	Male	10	Fall from height	Transverse fracture	LRA	Reconstruction plate + posterior column cannulated screw	39	< 1 mm	94	18
8	Female	14	Fall from height	Double column, bilateral Denis II sacral fracture	LRA	Reconstruction plate	29	< 1 mm	92	17
9	Female	14	Falls from height	Transverse fractures, unilateral saroiliac joint diastasis	LRA	Reconstruction plate + posterior column cannulated screw	30	12 mm	85	15
10	Female	13	Bicycle accidents	Anterior column with posterior hemi‐transverse	LRA	Reconstruction plate	32	4 mm	75	15
11	Male	14	Motorcycle accidents	Double column	LRA	Acetabular integrated anatomical plates	26	< 1 mm	90	18
12	Female	13	Falls from height	Double column, unilateral sacroiliac joint dislocation, pubic symphysis injury	LRA	Anterior + posterior column cannulated screw	24	7 mm	89	17
13	Female	14	Falls from height	Transverse fracture	LRA	Anterior column plate + posterior column screw	24	5 mm	91	18
14	Female	10	Car accidents	Transverse fracture	LRA	Anterior column plate + posterior column screw	13	< 1 mm	94	18

Abbreviation: LRA, lateral rectus approach.

### 2.4 Postoperative Management and Evaluation

Patients were immobilized for 2 weeks postoperatively and were encouraged to begin gentle passive hip joint range of motion exercises immediately after surgery under the supervision of a physiotherapist. At 6 weeks postoperatively, active range of motion exercises were started. Active strengthening partial weight‐bearing exercises were initiated at 3 months postoperatively. Normal activity and sports exercises were progressively allowed at 6 months postoperatively, and normal activity and sports exercises were allowed after bone healing. Each patient was followed up at 1, 2, 3, 6, and 12 months postoperatively, and yearly thereafter. Radiographs and clinical evaluations were performed at each follow‐up; the immediate postoperative images (X‐Ray and CT Scan) of fracture reduction were evaluated using Matta criteria while the functional outcome was assessed with the Modified Merle d'Aubigné and Postel scoring system which evaluates pain, gait, and mobility, on a scale of 1 to 6 for each item, where 1 indicates the worst and 6, the best state of the patient (the total minimum score reached is 3, and the maximum is 18); and the Harris Hip Score (HHS) where poor outcome refers to HHS < 70, fair 70–79, good 80–89, and excellent 90–100.

### 2.5 Statistical Analysis

Descriptive statistic was chosen as method of analysis; continuous variables were expressed as mean ± standard deviation (SD) while numbers were described as percentage. Statistical evaluation of the data was carried out using SPSS Statistics for Windows, version 27.0 (IBM Corp., Armonk, NY, USA).

## 3 Results

### 3.1 Basic Patients Clinical Data

From January 2019 to January 2025, 14 pediatric acetabular fracture patients were treated at our institution. All patients, including five males and nine females, with a mean age of 11.42 ± 2.24 years (Table 1) had a follow‐up period of longer than 12 months, mean 35.14 ± 12.66 months (Table 2); no patient was lost during follow‐up. In total, nine patients were injured on the left side, and five were injured on the right side. The mechanisms of injury essentially resulted from high‐velocity trauma and included car accidents *n* = 4, falls from height *n* = 8, motorcycle accidents *n* = 1, and bicycle accidents *n* = 1. The fracture classification distribution consisted of eight double column, five had transverse, and one anterior with posterior hemi‐transverse fracture according to Judet and Letournel classification of acetabular fractures. Concomitant posterior pelvic ring fracture with unilateral sacroiliac joint disruption was observed in five cases; the overall time from injury to surgery averaged 11.5 days, with a standard deviation of 13.36 days.

### 3.2 Operative Management and Functional Outcomes

In this study, all patients were treated operatively; among these patients who underwent operative treatment, eight patients were managed with reconstruction plates, two were fixed using anterior column plate + posterior column screw, three were fixed with anterior column screw and one case was managed with acetabular integrated anatomical plates; each patient achieved bone union during follow‐up, and 10 patients underwent internal fixation (plate, screw) removal after bone healing. With a mean final follow‐up of 33.71 ± 14.41 months (range, 13–56 months), at final follow‐up, the mean Harris Hip Score was 90.35 ± 5.58 points (range, 75–97 points), reported as 10 excellent, three good, one case with fair grade and the mean Merle d’Aubigné score was 17.21 ± 1.12 points (range, 15–18 points).

In this series, operative management was delayed (> 3 weeks post‐injury) in 3 of 14 patients (21.4%). The delays were 6 weeks for one patient (Case 8) and 8 weeks for the remaining two (Cases 9 and 10).

Despite the delay, all three patients achieved favorable functional outcomes at final follow‐up. Their Harris Hip Scores were 92 (excellent), 85 (good), and 75 (fair), with corresponding Merle d'Aubigné scores of 17, 15, and 15, respectively. Notably, while the immediate postoperative reduction quality (per Matta's criteria) varied among these delayed cases, it did not preclude a good clinical result, underscoring the feasibility of successful surgical intervention even in subacute presentations.

### 3.3 Complications

Three patients experienced mild, transient hip pain postoperatively. The pain was managed effectively with a regimen of anti‐inflammatory and analgesic medications, combined with temporary weight‐bearing restrictions during physical therapy. All three patients reported complete resolution of symptoms within 6 months; there were no other traumatic related complications such as posttraumatic osteoarthritis, osteonecrosis, joint dysplasia, leg length discrepancy, nonunion, and postoperative infection.

## 4 Discussion

The management of pediatric acetabular fractures remains guided by limited and predominantly historical literature, highlighting a significant gap in modern, evidence‐based surgical protocols. Most reports combine these fractures with pelvic ring injuries in about 68% of cases [5, 14], primarily focusing on pelvic trauma, associated injuries, and potential life‐threatening complications. Large series of pediatric acetabular fractures are uncommon; Tomaszewski et al. [2] reported a series of nine patients over a period of 20 years; Liu et al. [1] recently reported a case of a 13‐year‐old boy with a posterior column acetabular fracture; our study reports a series of 14 patients over a period of 5 years, outlining that acetabular injury in children is becoming common. Management of these patients can interfere with the presence of serious associated injuries, thus affecting the final clinical outcome. The main strength of the current study is that it does not only report an updated series of pediatric patients with complex acetabular fractures, it also demonstrates that even in patients presenting with old fractures, delayed operative management can still provide satisfactory clinical outcome.

### 4.1 Principal Research Findings

This study reports successful outcomes in a contemporary series of 14 pediatric acetabular fractures managed with a consistent surgical protocol using the lateral rectus abdominis approach and internal fixation. The protocol achieved excellent functional results, with a mean Harris Hip Score of 90.35 ± 5.58, a mean Merle d'Aubigné score of 17.21 ± 1.12, and a 100% union rate without major complications. Critically, it demonstrates that delayed surgical intervention (> 3 weeks post‐injury) in 21.4% of patients, often due to polytrauma, did not preclude favorable mid‐term outcomes, challenging the paradigm of mandatory early surgery. The strategy emphasized physeal preservation through careful implant placement and its removal, utilized a standardized surgical approach, and incorporated safe perioperative management, resulting in a low complication rate and no observed growth disturbances. Due to the rarity of pediatric acetabular injuries [5, 15, 16], the present report seems to be the only one reporting a larger series of patients presenting with such injury in this population group in the last decade. The results of our study demonstrated that in children, complex acetabular fractures can be managed operatively even in patients with delayed care, and present excellent results.

### 4.2 Surgical Management and Outcomes

Previous studies had discussed and recommended different approaches to the management of acetabular fractures in children. According to previous authors, in younger patients with ongoing growth, fixation techniques that do not cross the physis anatomically may be used [5]. In our series, all fractures were managed surgically and the fixation option of choice was plate and screw. The postoperative clinical variables indicated excellent outcomes; the Harris Hip Score reported 10 excellent, three good, and one case with fair score; while the mean Merle d’Aubigné score was 17.21 ± 1.12 points (range, 15–18 points). In our cohort, three cases of mild post‐operative hip pain were observed; however, the underlying etiology remained poorly understood. Tomaszewski et al. [2] in their series of nine patients, the average Merle d’Aubigné score at 12 months post‐treatment was 17.1 points, with a range of 13 to 18 points; several authors have reported small case series of operatively managed injuries (Table 3). While these reports lack consistent long‐term follow‐up data, all documented favorable short‐term outcomes at the time of hospital discharge [1, 17, 18, 19]. The findings of our study were basically consistent with that of the above authors. In our opinion, surgical intervention is indicated for all displaced acetabular fractures, including pediatric patients, to restore articular congruity. The selection of an internal fixation construct must be tailored to the specific fracture pattern and displacement. However, in pediatric patients, particular attention must be paid to the physis (growth plate). The plate should be positioned to avoid damaging the triradiate cartilage, and their timely removal should be considered once fracture healing is complete to mitigate the risk of iatrogenic growth disturbance.

**TABLE 3:** Literature review on Pediatric acetabular fractures.

Authors	years	Number of cases	Patients' ages (years)	Fracture type	Management approach	Clinical outcome
Hearty et al. [16]	2011	2	11	Posterior wall	Surgical treatment, posterior‐lateral approach	Not clearly reported
			8	Posterior wall	Surgical treatment, posterior‐lateral approach	Lost to follow‐up
Khair et al. [17]	2014	1	8	Posterior wall	Surgical treatment, posterior approach	Excellent clinical and radiographic results, return to normal including sport activities
L. Lucio et al. [15]	2015	1	12	Torode and Zieg IVd	Surgical management, Ilioinguinal approach	Short follow‐up (1 month), absence of post discharge pain at the hip joint.
Tomaszewski et al. [2]	2021	9 (4girls, 5 boys)	Mean 14.5 (range, 12–16.5)	Complex acetabular fractures	Surgical treatment in six patients and two were managed conservatively.	Mean Merle d'Aubigne score at 12 months after treatment was 17.1 points
Liu et al. [1]	2023	1	13	Posterior column	Surgical treatment, K‐L approach	Not reported

Abbreviation: K‐L, Kocher‐Langenbeck approach.

### 4.3 Analysis of Delayed Versus Early Intervention: A Case‐Based Comparison

**FIGURE 2:**
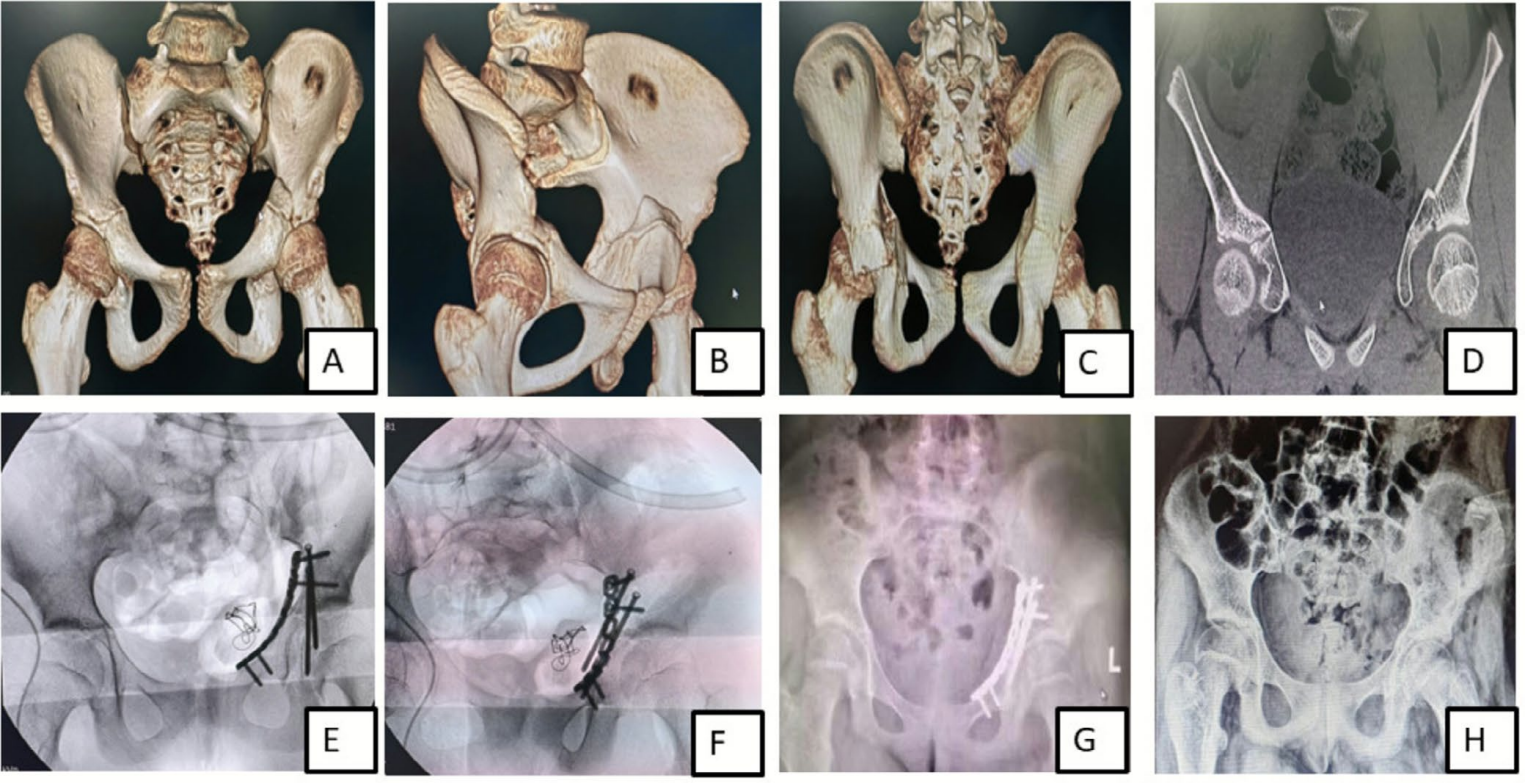
A 10‐year‐old female sustained a fall resulting in a transverse fracture of the left acetabulum and underwent surgery 5 days after the injury. CT 3D reconstruction images include the AP view (A), left oblique iliac view (B), posterior view (C), and coronal view (D), showing a transverse fracture of the left acetabulum. The posterior column was fixed with two screws, and the anterior column was reconstructed with a steel plate placed across the “Y” cartilage area (E and F). Postoperative radiograph in the AP view (G) confirmed that the fracture was reduced, fixed, and acetabular congruency restored. One year post‐surgery, the X‐ray (H) showed satisfactory fracture healing, and the internal fixation was removed.

**FIGURE 3:**
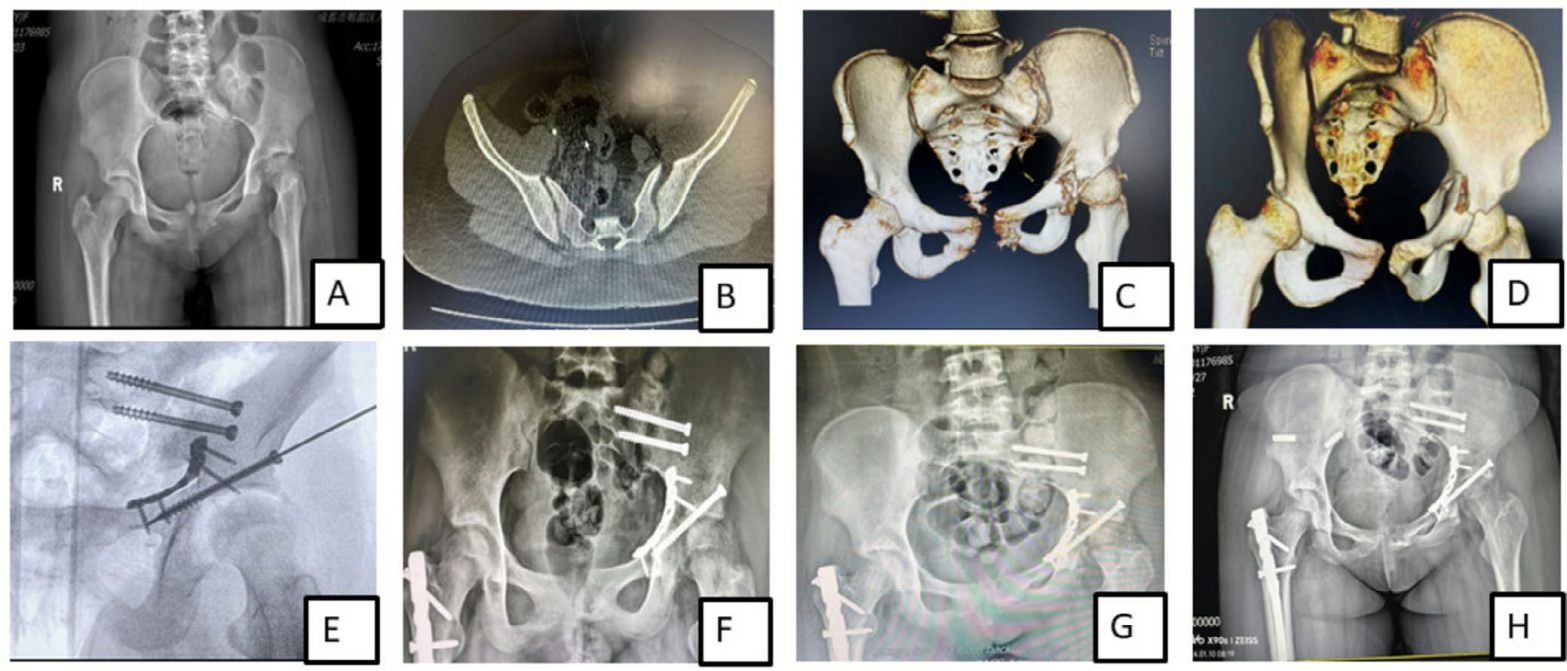
A 14‐year‐old female patient sustained an acetabular fracture after falling from a height. The post‐traumatic radiograph (AP view) showed a left acetabular fracture, left sacroiliac joint diastasis and right femoral shaft fracture (A). Preoperative CT scans revealed left sacroiliac joint disruption (B) and a minor pubic symphysis injury (C). 3D CT images from the left iliac view demonstrated a transverse fracture of the left acetabulum (D). Intraoperative fluoroscopic image displayed the fixation approach (E). Postoperative follow‐up radiographs were taken at 1 month (F), 6 months (G), and 1 year (H), respectively, showing good healing progression.

A retrospective study conducted by Matta et al. [20] assessed the outcomes of delayed surgical intervention for acetabular fractures in 61 patients. The results indicated that delayed surgery was associated with a higher risk of postoperative complications, including malunion, nonunion, and infection as compared to early definitive surgical intervention. In our series, we compared two cases with similar fracture patterns, operated at different time intervals after injury: they were one female patient (case 7, Table 2), 10 year‐old, who sustained transverse fracture of the acetabulum and was operated 5 days following injury (Figure 2) and a second patient (case 9), 14 year‐old female patient presenting with an old fracture (2 months) of the same pattern (Figure 3), who due to associated injuries, was suggested for conservative treatment in an external hospital; both patients were managed surgically at our center; the surgical intervention of the first patient was relatively simple and consisted of regular open reduction and internal fixation while in the second case, intraoperative osteotomy followed by articular surface reconstruction was carried out. The immediate postoperative Matta's criteria of fracture reduction showed that anatomic reduction (< 1 mm) was achieved in the first patient (case 7) while there was imperfect reduction (3 mm) in the second patient (case 9); however the operation was more complicated in case 9 (old fracture) than in case 7 (fresh fracture), the patient's clinical outcome at final follow‐up was good; the Harris Hip Score and Merle d’Aubigné score were good, respectively 85 and 15, while in the former case, these scores were excellent 94 and 18 respectively with full return to normal activity after 1 year following operation in both cases. Overall, these findings suggested that in the conditions of appropriate medical facilities and experienced surgical team, even old acetabular fractures can be operatively addressed and yield good clinical outcomes; however, whether the positive result of these typical cases was related to the skeletal immaturity or the surgical team's ability was not clearly understood and yet to be demonstrated. Additionally, due to the limited number of cases, the comparison of the postoperative clinical outcome could not result in relevant statistically significant data; therefore, larger numbers of similar cases are required to approve this hypothesis. We believe that, irrespective of the fracture's age, all attempts should be explored to achieve anatomical congruity of the articular surface.

### 4.4 Impact of Polytrauma on Surgical Timing

In this series, delayed definitive surgery for the acetabular fracture was most often necessitated by the priority management of severe, life‐threatening concomitant injuries. The 14‐year‐old female adolescent patient described in Figure 1 was admitted to the hospital following a high‐altitude fall from the fifth floor. Upon admission, the patient was diagnosed with bilateral comminuted fractures of the femurs and the left femoral condyle, as well as intracranial hemorrhage. Emergency surgical intervention was performed, including debridement, fracture reduction, external fixation, and mechanical ventilation support to stabilize the patient's condition. The patient remained comatose for approximately 1 month before regaining consciousness. Following extubation, the patient developed a pulmonary infection and was admitted to the intensive care unit (ICU) for a total of 45 days before being transferred to the orthopedic department for further management. The above condition delayed the fracture management for 2 months. The condition has been described by some authors [5, 21] and unfortunately remains a non‐modifiable factor influencing early total care in this patient group.

### 4.5 Critical Factors Influencing Long‐Term Outcome

The role of acetabular articular surface incongruence in the development of posttraumatic complication is not a new topic of discussion; Heeg et al. [22] proposed that the congruency of the reduction is a key factor in determining both the clinical and radiological outcomes; the author's approach of open reduction and internal fixation successfully achieved acetabular congruency in 13 out of 16 acetabular fractures, with 11 patients showing satisfactory results. According to other authors, achieving and preserving hip congruency through closed reduction has been reported to be challenging and, in some cases, unfeasible [23]. In this study, all fractures with displacement greater than 2 mm were managed surgically to restore joint congruency. Although some cases did not achieve excellent anatomical joint congruency integrity on immediate postoperative radiographs according to Matta criteria (2–4 mm as seen in Table 1), they still presented with good clinical and functional outcome on final follow‐up (Figure 1, Patient 8 in Table 1).

Another important key factor for achieving postoperative good functional outcome is the timing of rehabilitation. Following rules, we recommended early postoperative joint passive follow with active exercise to improve joint mobility and achieve early full range of motion of the joint (Figure 1). Additionally, it is known that the pattern of pediatric acetabular fracture may disrupt the growth plate (triradiate cartilage); on the other hand, its fixation with plate may equally interfere with the development of the pelvis and the articular surface. Acetabular fractures involving triradiate cartilage damage, premature closure can result in acetabular dysplasia, potentially leading to femoral head subluxation; this type of dysplasia occurs in fewer than 5% of acetabular fracture cases [4]. Complications following triradiate cartilage injury, such as growth arrest, may lead to hip dysplasia and leg length discrepancy [24]. It is recommended to remove any internal fixation device crossing the physis once healing is complete [25]; therefore, having known the risk of premature physeal fusion and the formation of a growth arrest related to the child's age at time of injury, we strongly recommend to our patients to proceed with plate removal at 1 year following the operation to improve long‐term postoperative outcome; fortunately, none of our patients in the current series developed leg length discrepancy, even those who presented with old fractures associated with shortness (about 3 cm) of the leg such as in Figure 1; one of our targets in this patient was to achieve similar leg length in both limbs. In our opinion, we believe that, to overcome the dilemma between the fracture healing duration and the risk of growth disturbance, 1 year time is reasonable to remove the internal fixation device. We shall acknowledge that this question is still open for discussion. Future research on this population group will thoroughly reevaluate this concern and draw appropriate conclusion.

### 4.6 Treatment Considerations by Skeletal Maturity

In older children with closed triradiate cartilage, the treatment of acetabular fractures follows the same approach as in adults with a few key differences [25]. Surgery is considered only in cases where inter‐fragment displacement exceeds 2 mm or when there is joint instability [5]. In children with open growth plates, the primary goal is to preserve acetabular growth to minimize long‐term functional impacts on the hip [26]. According to Tomaszewski et al. [2], the treatment of acetabular fractures in pediatric patients during or after puberty may be similar to that in adults; in our series, all patients were at puberty age or younger; they were all managed operatively with excellent outcomes. All fractures successfully healed without any signs of infection, fixation failure, or nonunion. At the final follow‐up, three patients (21.4%) experienced hip pain, and one exhibited restricted hip range of motion. Due to the relatively brief follow‐up period, posttraumatic arthritis and/or chondrolysis may not have yet been revealed. These findings were consistent with current literature [5]. We believe that our approach, even if not aligning with the existing literature, will contribute in opening a new research orientation on the treatment of these fractures.

### 4.7 Summary of Surgical and Management Strategies

This study validates a valuable, pediatric‐specific surgical strategy for acetabular fractures, centered on the consistent use of the lateral rectus abdominis approach and plate‐screw fixation.

The protocol's efficacy was demonstrated by excellent functional outcomes (mean HHS: 90.35, mean Merle d'Aubigné: 17.21) and a 100% union rate. A central and novel finding is the successful management of delayed cases (> 3 weeks post‐injury), challenging the mandate for early surgery, often necessitated by concomitant polytrauma. The strategy prioritizes physeal preservation through careful implant placement and planned their removal at one year, resulting in no growth disturbances. This approach proves that anatomical restoration and good results are achievable even in delayed presentations when performed by an experienced team.

### 4.8 Study Limitations

The main limitations are the retrospective, single‐center design, limited follow‐up, and absence of a control group, all of which are potential sources of bias. However, given the rarity of these injuries, the objective was not to compare different treatment approaches but rather to present our cases, outline our management strategy, and demonstrate that even patients with delayed surgical intervention can achieve satisfactory outcomes. Additionally, our surgical team was composed of a main surgeon, with a consistent group of assistant surgeons. We acknowledge that having a single main surgeon may introduce a potential bias in the consistency of surgical decision‐making, particularly regarding the choice of approach. While we sought to contextualize our findings within the existing literature, a direct comparison was limited by a scarcity of published data with a large number of cases managed using similar methodology. Future multi‐center studies with larger patient cohorts and extended follow‐up are needed to validate these findings and enable a direct comparison of clinical outcomes between early and delayed operative management.

## 5 Conclusions

Acetabular fractures in children are rare and typically result from high‐energy trauma. Treatment should focus on achieving optimal postoperative outcomes, with an emphasis on anatomical reduction, even in cases of delayed presentation. The findings of this study indicate that, in specialized medical centers with experienced surgical teams, patients undergoing delayed surgical management can still achieve anatomical reduction and satisfactory clinical outcomes.

## Author Contributions

Conceptualization: Guy Romeo Kenmegne and Shicai Fan. Data curation: Guy Romeo Kenmegne, Yuqing Wang, Wentong Zhao, and Rui Zeng. Formal analysis: Ziming Zhang, Yuqing Wang, Wentong Zhao, Jiafu Miu, and Yilan Liao. Methodology: Guy Romeo Kenmegne, Rui Zeng, Gang Ma, Sheqiang Chen, and Qiyan Zhou. Validation: Kai Zeng. Investigation: Guy Romeo Kenmegne. Writing original draft preparation: Guy Romeo Kenmegne. Visualization: Sheqiang Chen and Qiyan Zhou. Supervision: Shicai Fan. All authors reviewed the final version of the manuscript.

## Funding

This work was supported by the National Key Research and Development Program of China (2022YFC2504303), the Guangzhou Clinical High‐tech and Major Technology Projects (2024PL‐GX11), and the National Natural Science Foundation of China (82072411).

## Ethics Statement

This study was approved by the Ethical Committee of the hospital (no. 202104008), date 2021‐04‐30 and performed in accordance with the 1964 Helsinki Declaration and its later amendments or comparable ethical standards.

## Consent

Written informed consent was obtained from all participating patients' legal guardians, with specific authorization for both study participation and the use of radiographic/clinical images in scientific publications.

## Conflicts of Interest

The authors declare no conflicts of interest.

## Data Availability

The data that support the findings of this study are available on request from the corresponding author. The data are not publicly available due to privacy or ethical restrictions.
